# Standing Wave Binding of Hemispherical Resonator Containing First–Third Harmonics of Mass Imperfection under Linear Vibration Excitation

**DOI:** 10.3390/s20195454

**Published:** 2020-09-23

**Authors:** Yan Huo, Shunqing Ren, Zhennan Wei, Guoxing Yi

**Affiliations:** Space Control and Inertial Technology Research Center, Harbin Institute of Technology, Harbin 150080, China; yhuo@hit.edu.cn (Y.H.); wzn@hit.edu.cn (Z.W.); ygx@hit.edu.cn (G.Y.)

**Keywords:** hemispherical resonator, mass imperfection, motion equation, linear vibration, standing wave binding

## Abstract

Due to complicated processing technology, the mass distribution of a hemispherical resonator made of fused silica is not uniform, which can affect the azimuth of the standing wave of a resonator under the linear vibration excitation. Therefore, the analysis of standing wave evolution of a resonator with mass imperfection under linear vibration excitation is of significance for the improvement of the output accuracy of a gyroscope. In this paper, it is assumed that the resonator containing the first–third harmonics of mass imperfection is excited by horizontal and vertical linear vibration, respectively; then, the equations of motion of an imperfect resonator under the second-order vibration mode are established by the elastic thin shell theory and Lagrange mechanics principle. Through error mechanism analysis, it is found that, when the frequency of linear vibration is equal to the natural frequency of resonator, the standing wave is bound in the azimuth of different harmonics of mass imperfection with the change in vibration excitation direction. In other words, there are parasitic components in the azimuth of the standing wave of a resonator under linear vibration excitation, which can cause distortion of the output signal of a gyroscope. On the other hand, according to the standing wave binding phenomenon, the azimuths of the first–third harmonics of mass imperfection of a resonator can also be identified under linear vibration excitation, which can provide a theoretical method for the mass balance of an imperfect resonator.

## 1. Introduction

Hemispherical resonators composed of fused quartz were studied extensively by researchers all over the world, especially for their engineering applications, such as a hemispherical resonance gyroscope (HRG). An HRG [1,2,3] is a kind of all-solid vibratory gyros based on the Coriolis effect, well known for its high precision, high reliability, simple mechanical structure, long service life, low power consumption, miniaturization, and radiation resistance. Thus, it is particularly suitable for long-term space missions; for example, the National Aeronautics and Space Administration (NASA) applied an HRG for the Hubble Space Telescope and Cassini spacecraft [4]. However, the exceptional performance of an HRG is limited by the imperfections of the resonator, including mass, stiffness, and damping imperfections, where the mass and stiffness imperfections lead to a mismatch between the natural frequencies of the two principal vibration modes of the resonator, which is one of the error sources of the gyroscope. In order to analyze the dynamics of a Coriolis vibratory gyroscope (CVG), Lynch [5] established the generalized equations of motion of a CVG including frequency and damping imperfections of the resonator, which are the most widely used and are being written into IEEE standards [6]. Moreover, Lynch also derived averaged equations which can describe the dynamics of a resonator including the influence of control loops, which provides a theoretical basis for the design of the control system of a CVG. Loveday and Rogers [7] established a system of averaged equations describing the dynamics of a CVG and designed the force-to-rebalance control system to eliminate the first-order effects of frequency imperfections of a resonator. Fox [8] considered the flexural radial vibration of circular rings with imperfections represented by small attached masses and springs, introduced the concept of “equivalent imperfection mass”, and proposed a method of eliminating the frequency split by removing mass from the antinode of the low-frequency axis or by adding mass to the antinode of the high-frequency axis. Choi and Kim [9] pointed out that the effect of point masses on a hemispherical resonator can be expressed by functions for the frequency split and shift angle of the vibration mode, while the dynamic model of an imperfect resonator with multiple point masses can be represented by the model of an imperfect resonator with an equivalent single-point mass. Basarab et al. [10] proposed a chemical etching method to remove the first–fourth harmonics of mass nonuniformity of an imperfect resonator and gave the rotation angle about the axis of symmetry of resonator, the depth and inclination of resonator immersion into a chemical bath, and the duration time of chemical etching of resonator. Wang et al. [11] investigated a chemical etching procedure to remove the fourth harmonic of mass nonuniformity of a hemispherical resonator, which resulted in a frequency split, and the experimental results indicated that the frequency split of the resonator could be reduced to 0.05 Hz. Gallacher et al. [12] demonstrated that the nonlinear negative spring component produced from a particular arrangement of capacitive electrodes is capable of removing the mass and stiffness imperfections. Schwartz et al. [13] proposed a mass matrix perturbation method for the frequency tuning of a disc resonator gyroscope, where small magnets were used to induce perturbations of resonator. Xiao et al. [14] presented the electrostatic balance method to reduce the frequency split of a disc resonator gyroscope, and the experimental results indicated that the frequency split of resonator could be less than 0.03 Hz. Senkal et al. [15] demonstrated the sub-1 Hz frequency symmetry in micro-glassblown wineglass resonators with integrated electrode structures for the first time and proposed a new fabrication process based on deep glass dry etching to fabricate micro wineglasses with self-aligned stem structures and integrated electrodes. In [16], the hemispherical resonator was equivalent to a thin elastic ring, and the relationship between the frequency split and the fourth harmonic of mass nonuniformity was deduced, which could be used to calculate the magnitude of trimming mass. Shao et al. [17] reported an integrated polysilicon micro-hemispherical resonator gyroscope with self-aligned drive, sense, and tuning electrodes, all fabricated using a single-wafer process. Xi et al. [18] presented an acoustic method to measure the frequency split, quality factor, and deflection of vibration mode of a resonator using the superposition and decomposition of acoustic waves generated by standing wave eigenmodes of the resonator. Hu et al. [19] used the ion beam to remove the first–fourth harmonics of mass imperfection of a hemispherical resonator, and the experimental result indicated that the frequency split was reduced from 0.46 Hz to 0.004 Hz. Wan et al. [20] reported a highly symmetry polysilicon microscale HRG with spherical electrodes, and the frequency split of the resonator could be reduced from 10 Hz to 0.51 Hz by tuning the voltages of electrodes. Tao et al. [21] established a dynamic model of a lumped mass of a cupped vibratory gyroscope using the discretization method, analyzed the effects of different position trimming of the imperfect resonator on the frequency split, and then developed a balance method based on cup-bottom trimming, giving the entire procedures for frequency trimming of the resonator; the finite element method simulation results indicated that the frequency split could be reduced to 10^−3^ Hz. Abdelmoneum et al. [22] demonstrated geometrically symmetrical laser targeting in maintaining high quality on the microscale, much higher than on the macroscale for different types of micromechanical resonators. Kim and Kim [23] deduced the analytical model of frequency split on a hemispherical shell with mass imperfection, and they established a function to predict the trimming mass of an imperfect resonator. Xi et al. [24] analyzed the stability of a vibration system using the imperfect ring model, and the theoretical analysis results indicated that the offset of the principal vibration axis, which gives rise to the bias of an all solid-state vibratory gyroscope, is affected synthetically by the frequency split, exciting force, and damping coefficient. Wang et al. [25] designed an FPGA digital circuit of force rebalance control based on the equations of motion of an HRG containing a frequency split and damping nonuniformity, and the experiment indicated that the HRG had a higher linearity scale factor and lower bias stability. Zhao et al. [26] established the equations of motion of a resonator with density imperfection using the Bubonov–Galerkin method, and they derived a drift model in a short time by applying the averaging method. Tao et al. [27] proposed a method for the identification of the mode offset of a cupped vibration gyroscope based on the output signal detection of a piezoelectric electrode. In the above references, the mass imperfection of a resonator was equivalent to the mass point instead of a harmonic form, which led to some error mechanism problems unable to be analyzed. In comparison, the advantage of a Fourier series is to orthogonalize the mass imperfection and analyze the influence of each harmonic component on the vibration characteristics of a resonator. Huo [28] deduced that the fourth harmonic of mass imperfection has the greatest influence on the frequency split. Due to the orthogonality of the trigonometric function system and the second-order vibration of a resonator, the first–third harmonics of mass imperfection cannot appear in the equations of motion of an imperfect resonator. However, when the resonator is in a linear vibration environment, the influence of the first–third harmonics of mass imperfection on the azimuth of a standing wave cannot be ignored, which is the focus of this paper. 

In this paper, the mass imperfection is expressed in the form of a Fourier expansion of density with respect to the circumference direction of the resonator; and the equations of motion of a resonator containing the first–third harmonics of mass imperfection are established using the theory of elastic thin shell and the Lagrange mechanics principle. Moreover, the inertial force caused by the external linear vibration excitation is considered. Based on the equations of motion, the binding phenomenon of a standing wave is analyzed. On the other hand, according to the binding phenomenon of a standing wave, an identification method for the location of the first–third harmonics of mass imperfection is proposed, and the numerical simulation model based on the equations of motion of an imperfect resonator proves the correctness of the identification method.

## 2. Equations of Motion of Hemispherical Resonator with First–Third Harmonics of Mass Imperfection

### 2.1. Basic Structure of HRG

**Figure 1:**
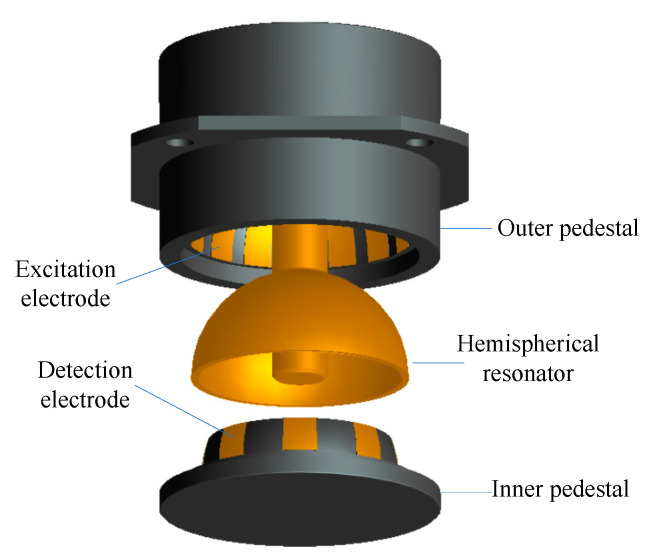
Structure of a hemispherical resonance gyroscope (HRG).

**Figure 2:**
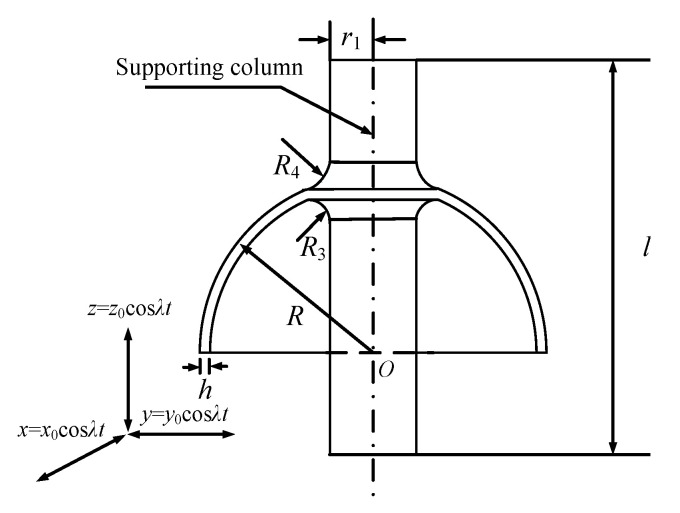
Sectional view of a hemispherical resonator.

The sensitive element of HRG in force feedback mode is composed of three parts: outer pedestal, hemispherical resonator, and inner pedestal, as shown in Figure 1. Sixteen excitation electrodes are evenly distributed on the inner surface of the outer pedestal, which are used for the amplitude, quadrature, and rate control of resonator. Eight detection electrodes are evenly distributed on the outer surface of the inner pedestal, which are used to detect the frequency, amplitude, azimuth of the standing wave, and orthogonal vibration component of the resonator. The geometric structure of the resonator is shown in Figure 2.

In this section, the equations of motion of a hemispherical resonator containing the first–third harmonics of mass imperfection under the second-order vibration mode are established using the Lagrange mechanics principle and the theory of elastic thin shell.

### 2.2. Establishment of Coordinate Systems of Hemispherical Resonator

**Orthogonal Curvilinear Coordinate System***P*-*αβz*: Origin *P* is located on the mid-surface of the resonator, axis *z* is the normal line of the mid-surface, and axes *α* and *β* are mutually orthogonal tangent lines located on the mid-surface.

**Hemispherical Resonator Coordinate System***O*-*x_h_y_h_y_h_*: Origin *O* is at the center of the ground opening of the resonator. Axis *z_h_* is coincident with the symmetry axis of the resonator. Axes *x_h_* and *y_h_* are perpendicular and lying on the ground opening plane of the resonator.

**Local Coordinate System***P*-*e*_1_*e*_2_*m*: Origin *P* is located on the mid-surface of the resonator, while axes *e*_1_, *e*_2_, and *m* are parallel to the tangential lines *α*, *β*, and *z*, respectively.

**Rotating Coordinate System***O*-*x_r_y_r_z_r_*: Origin *O* is at the center of the ground opening of the resonator, while axes *x_r_*, *y_r_*, and *z_r_* are parallel to the axes *e*_1_, *e*_2_, and *m*, respectively.

The attitude matrix from *O*-*x_h_y_h_z_h_* to *P*-*e*_1_*e*_2_*m* is described as

(1) $$C_{h}^{e}=\left[\begin{array}{ccc} \cos\alpha & 0 & -\sin\alpha \\ 0 & 1 & 0\\ \sin\alpha & 0 & \cos\alpha \end{array}\right]\left[\begin{array}{ccc} \cos\beta & \sin\beta & 0\\ -\sin\beta & \cos\beta & 0\\ 0 & 0 & 1 \end{array}\right]=\left[\begin{array}{ccc} \cos\alpha \cos\beta & \cos\alpha \sin\beta & -\sin\alpha \\ -\sin\beta & \cos\beta & 0\\ \sin\alpha \cos\beta & \sin\alpha \sin\beta & \cos\alpha \end{array}\right].$$

**Figure 3:**
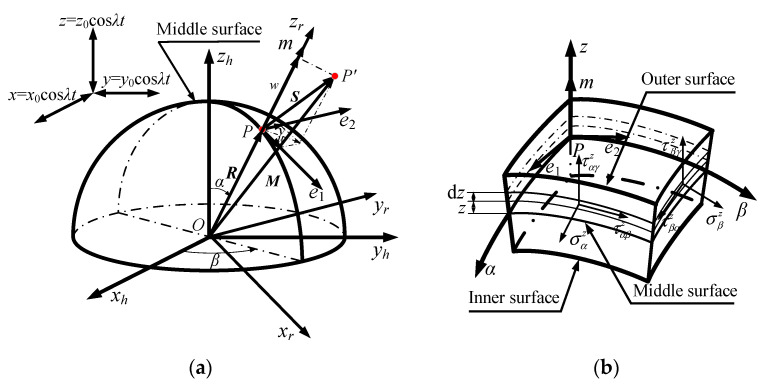
(**a**) Mid-surface deformation of a hemispherical resonator; (**b**) Stress of hemispherical thin shell microelement.

The mid-surface deformation and shell stress of a hemispherical resonator are shown in Figure 3a,b. In addition, the defined coordinate systems are also shown in Figure 3a,b. 

### 2.3. Deformation Energy of Hemispherical Resonator

The deformation equations of the mid-surface of a hemispherical resonator are obtained as follows [29]:

(2) $$\{\begin{array}{lll} \varepsilon_{\alpha }=\frac{1}{R}\left(\frac{\partial u}{\partial \alpha }+w\right) & \varepsilon_{\beta }=\frac{1}{R\sin\alpha }\left(\frac{\partial v}{\partial \beta }+u\cos\alpha +w\sin\alpha \right) & \gamma_{\alpha \beta }=\frac{1}{R}\left(\frac{\partial v}{\partial \alpha }+\frac{1}{\sin\alpha }\frac{\partial u}{\partial \beta }-v\cot\alpha \right)\\ \kappa_{1}=-\frac{1}{R^{2}}\left(\frac{\partial^{2}w}{\partial \alpha^{2}}+w\right) & \kappa_{2}=-\frac{1}{R^{2}}\left(\frac{1}{\sin^{2}\alpha }\frac{\partial^{2}w}{\partial \beta^{2}}+\frac{\partial w}{\partial \alpha }\cot\alpha +w\right) & \chi =-\frac{1}{R^{2}\sin\alpha }\left(\frac{\partial^{2}w}{\partial \alpha \partial \beta }-\frac{\partial w}{\partial \beta }\cot\alpha \right) \end{array},$$

where *ε_α_* and *ε_β_* are the normal strains of the mid-surface in the directions *α* and *β*. *γ_αβ_* is the shear strain on the *αβ* plane determined by coordinate tangential lines *α* and *β*. *κ*_1_ and *κ*_2_ are the principal curvature of the mid-surface along the directions *α* and *β*. *χ* is the twist rate of the mid-surface. *R* is the curvature radius of the mid-surface, as shown in Figure 3.

The strains of the point with the displacement *z* from the mid-surface of a hemispherical resonator are expressed as follows [30]:

(3) $$\{\begin{array}{l} \varepsilon_{\alpha }^{z}=\varepsilon_{\alpha }+\kappa_{1}z+O_{\alpha }^{z}\left(z^{2}\right)\\ \varepsilon_{\beta }^{z}=\varepsilon_{\beta }+\kappa_{2}z+O_{\beta }^{z}\left(z^{2}\right)\\ \gamma_{\alpha \beta }^{z}=\gamma_{\alpha \beta }+2\chi z+2O_{\alpha \beta }^{z}\left(z^{2}\right) \end{array}.$$

The relationships between stress and strain displacement *z* from the mid-surface of a hemispherical resonator by Hooke’s law [30] are given by

(4) $$\{\begin{array}{l} \sigma_{\alpha }^{z}=\frac{E}{1-\mu^{2}}\left(\varepsilon_{\alpha }^{z}+\mu \varepsilon_{\beta }^{z}\right)\\ \sigma_{\beta }^{z}=\frac{E}{1-\mu^{2}}\left(\varepsilon_{\beta }^{z}+\mu \varepsilon_{\alpha }^{z}\right)\\ \tau_{\alpha \beta }^{z}=\frac{E}{2\left(1+\mu \right)}\gamma_{\alpha \beta }^{z} \end{array},$$

where *E* and *μ* are the Young’s modulus and the Poisson’s ratio of the resonator, respectively. $\sigma_{\alpha }^{z}$ and $\tau_{\alpha \beta }^{z}$ are the normal stress and shear stress of the mid-surface in the *α* direction, respectively. Similarly, $\sigma_{\beta }^{z}$ and $\tau_{\beta \alpha }^{z}$ are the normal stress and shear stress of the mid-surface in the *β* direction, respectively, as shown in Figure 3b.

The deformation energy [30] of a hemispherical resonator is written as

(5) $$Q=\frac{1}{2}\int_{-\frac{h}{2}}^{\frac{h}{2}}\int_{0}^{2\pi }\int_{0}^{\frac{\pi }{2}}\left(\sigma_{\alpha }^{z}\varepsilon_{\alpha }^{z}+\sigma_{\beta }^{z}\varepsilon_{\beta }^{z}+\tau_{\alpha \beta }^{z}\gamma_{\alpha \beta }^{z}\right)R^{2}\sin\alpha d\alpha d\beta dz,$$

where *h* is the thickness of the resonator, as shown in Figure 2.

The vibration amplitude of a resonator under the second-order vibration mode is only a few micrometers. Thus, the assumption that the mid-surface is not stretchable can be applied; then,

(6) $$\varepsilon_{\alpha }=\varepsilon_{\beta }=\gamma_{\alpha \beta }=0.$$

Substituting Equations (2)−(4) into Equation (5), the approximate deformation energy of a hemispherical resonator is described as

(7) $$Q=\frac{Eh^{3}}{24\left(1-\mu^{2}\right)}\int_{0}^{2\pi }\int_{0}^{\frac{\pi }{2}}\left[\kappa_{1}^{2}+\kappa_{2}^{2}+2\mu \kappa_{1}\kappa_{2}+2\left(1-\mu \right)\chi^{2}\right]R^{2}\sin\alpha d\alpha d\beta .$$

### 2.4. Second-Order Vibration Mode Function

**Figure 4:**
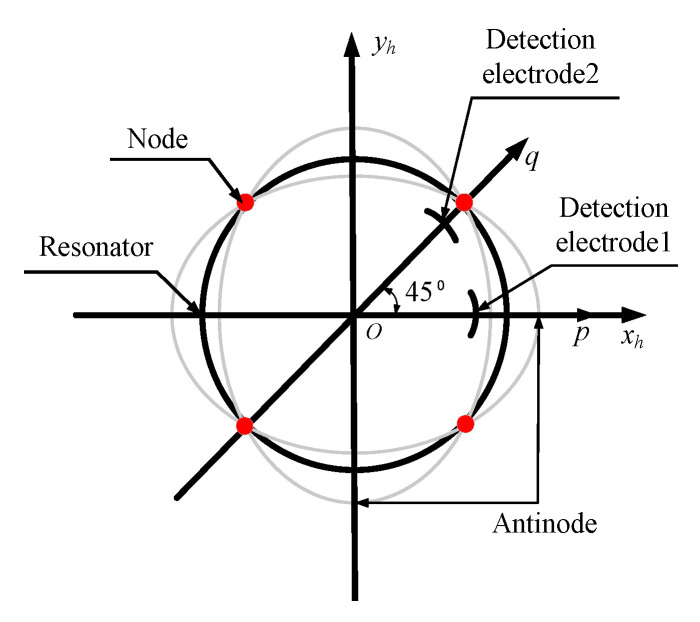
Second-order mode decomposition.

When the hemispherical resonator works in the second-order vibration mode, the displacements of a point on the mid-surface are denoted as follows [16]:

(8) $$\left[\begin{array}{c} u\left(\alpha ,\beta ,t\right)\\ v\left(\alpha ,\beta ,t\right)\\ w\left(\alpha ,\beta ,t\right) \end{array}\right]=\left[\begin{array}{l} U\left(\alpha \right)\cos2\beta \\ V\left(\alpha \right)\sin2\beta \\ W\left(\alpha \right)\cos2\beta \end{array}\right]p\left(t\right)+\left[\begin{array}{l} U\left(\alpha \right)\sin2\beta \\ -V\left(\alpha \right)\cos2\beta \\ W\left(\alpha \right)\sin2\beta \end{array}\right]q\left(t\right),$$

where *U*(*α*), *V*(*α*), and *W*(*α*) are Raleigh’s functions, which restrict the displacement relationship among different microelements of the resonator. *p*(*t*) and *q*(*t*) are time-dependent harmonic oscillation functions, which reveal the amplitude, frequency, and phase information of vibration, and their directions are shown in Figure 4.

Substituting Equation (8) into Equations (2) and (6), the differential equations are as follows:

(9) $$\{\begin{array}{l} W\left(\alpha \right)+\frac{\partial U\left(\alpha \right)}{\partial \alpha }=0\\ \frac{2V\left(\alpha \right)}{\sin\alpha }+U\left(\alpha \right)\cot\alpha -\frac{\partial U\left(\alpha \right)}{\partial \alpha }=0\\ \frac{2U\left(\alpha \right)}{\sin\alpha }+V\left(\alpha \right)\cot\alpha -\frac{\partial V\left(\alpha \right)}{\partial \alpha }=0 \end{array}.$$

By solving the differential equations, Raleigh’s functions are given by

(10) $$\{\begin{array}{c} U\left(\alpha \right)=V\left(\alpha \right)=C_{1}\sin\alpha \tan^{2}\left(\frac{\alpha }{2}\right)\\ W\left(\alpha \right)=-C_{1}\left(2+\cos\alpha \right)\tan^{2}\left(\frac{\alpha }{2}\right) \end{array}.$$

When central attention is paid to the form of Raleigh’s functions rather than their amplitudes, *C*_1_ = 1 can be considered.

### 2.5. Kinetic Energy of Hemispherical Resonator

When a hemispherical resonator works in the second-order vibration mode, point *P* on the mid-surface will move to $P^{\prime }$; the vector ***M*** in the rotating coordinate system *O*-*x_r_y_r_z_r_* is expressed as

(11) $$M={\left[\begin{array}{ccc} u & v & R+w \end{array}\right]}^{T}.$$

The absolute velocity of a point on the mid-surface of a hemispherical resonator is written as

(12) $$V=\frac{dM}{dt}={\left[\begin{array}{ccc} \dot{u} & \dot{v} & \dot{w} \end{array}\right]}^{T},$$

where $\dot{u}$, $\dot{v}$, and $\dot{w}$ are the first time derivatives of *u*, *v*, and *w*, respectively.

The approximate kinetic energy of a hemispherical resonator is obtained by

(13) $$T=\frac{1}{2}\int_{R-\frac{h}{2}}^{R+\frac{h}{2}}\int_{0}^{2\pi }\int_{0}^{\frac{\pi }{2}}V^{T}V\rho \left(\beta \right)r^{2}\sin\alpha d\alpha d\beta dr\approx \frac{1}{2}hR^{2}\int_{0}^{2\pi }\int_{0}^{\frac{\pi }{2}}\rho \left(\beta \right)\left({\dot{u}}^{2}+{\dot{v}}^{2}+{\dot{w}}^{2}\right)\sin\alpha d\alpha d\beta ,$$

where *ρ* is the density of the resonator, which is related to the circumferential angle.

The density *ρ* is expanded in the form of a Fourier series,

(14) $$\rho (\beta )=\rho_{0}\sum_{i=1}^{3}\left\{1+\varepsilon_{i}\cos\left[i\left(\beta -\beta_{i}\right)\right]\right\},$$

where *ρ*_0_ is average density, while *ε_i_* and *β_i_* are the relative amplitude and azimuth of the *i*-th harmonic of density imperfection, respectively.

### 2.6. Equations of Motion of Hemispherical Resonator Containing First–Third Harmonics of Mass Imperfection

It is known from the orthogonality of the trigonometric function system that, for integers *i* and *j*,

(15) $$\{\begin{array}{l} \int_{0}^{2\pi }\sin\left(i\beta \right)\sin\left(j\beta \right)d\beta =\{\begin{array}{c} 0\begin{array}{cc} & i\neq j \end{array}\\ \pi \begin{array}{cc} & i=j \end{array} \end{array}\\ \int_{0}^{2\pi }\cos\left(i\beta \right)\cos\left(j\beta \right)d\beta =\{\begin{array}{c} 0\begin{array}{cc} & i\neq j \end{array}\\ \pi \begin{array}{cc} & i=j \end{array} \end{array}\\ \int_{0}^{2\pi }\sin\left(i\beta \right)\cos\left(j\beta \right)d\beta =0\begin{array}{cc} & \mathrm{any}\;\mathrm{integer}\;i\;\mathrm{and}\;j \end{array} \end{array}.$$

Substituting Equations (8), (14), and (15) into Equation (13), the kinetic energy of a hemispherical resonator is rewritten as

(16) $$T=\frac{1}{2}I_{0}\left({\dot{p}}^{2}+{\dot{q}}^{2}\right),$$

where $\dot{p}$ and $\dot{q}$ are the first-order time derivatives of *p* and *q*, respectively. Similarly, $\ddot{p}$ and $\ddot{q}$ are the second-order time derivatives.

(17) $$I_{0}=\pi \rho_{0}hR^{2}\int_{0}^{\frac{\pi }{2}}\left[U^{2}\left(\alpha \right)+V^{2}\left(\alpha \right)+W^{2}\left(\alpha \right)\right]\sin\alpha d\alpha .$$

Applying Equations (8), (14), and (15) into Equation (7), the deformation energy of a hemispherical resonator is rearranged as

(18) $$Q=\frac{1}{2}I_{1}\left(p^{2}+q^{2}\right),$$

where

(19) $$\begin{array}{c} I_{1}=\frac{\pi Eh^{3}}{12\left(1-\mu^{2}\right)}\int_{0}^{\frac{\pi }{2}}\{\frac{\sin\alpha }{R^{2}}{\left[W\left(\alpha \right)+\frac{\partial^{2}W\left(\alpha \right)}{\partial \alpha^{2}}\right]}^{2}+\frac{\sin\alpha }{R^{2}}{\left[\left(1-\frac{4}{\sin^{2}\alpha }\right)W\left(\alpha \right)+\frac{\partial W\left(\alpha \right)}{\partial \alpha }\cot\alpha \right]}^{2}+\frac{2\mu \sin\alpha }{R^{2}}\cdot \\ \left[W\left(\alpha \right)+\frac{\partial^{2}W\left(\alpha \right)}{\partial \alpha^{2}}\right]\left[\left(1-\frac{4}{\sin^{2}\alpha }\right)W\left(\alpha \right)+\frac{\partial W\left(\alpha \right)}{\partial \alpha }\cot\alpha \right]+\frac{8(1-\mu )}{R^{2}\sin\alpha }{\left[\frac{\partial W\left(\alpha \right)}{\partial \alpha }-W\left(\alpha \right)\right]}^{2}\}d\alpha \end{array}$$

The Lagrange function of the second-order vibration mode of a hemispherical resonator is given by

(20) $$L\left(p,q,\dot{p},\dot{q}\right)=T-Q.$$

The second-order vibration mode is governed by the Lagrange equations

(21) $$\{\begin{array}{c} \frac{d}{dt}\left(\frac{\partial L}{\partial \dot{p}}\right)-\frac{\partial L}{\partial p}+\frac{\partial D}{\partial \dot{p}}=F_{p}\\ \frac{d}{dt}\left(\frac{\partial L}{\partial \dot{q}}\right)-\frac{\partial L}{\partial q}+\frac{\partial D}{\partial \dot{q}}=F_{q} \end{array},$$

where the energy dissipation function is

(22) $$D=\frac{1}{2}\left(\xi_{p}{\dot{p}}^{2}+\xi_{q}{\dot{q}}^{2}\right),$$

where *ξ_p_* = *ξ_q_* = *ξ* is the viscous damping coefficient of the hemispherical resonator. *F_p_* and *F_q_* are the external forces applied on the resonator. If the resonator is in an accelerated state, then

(23) $$F_{p}=F_{p-e}+F_{p-i},F_{q}=F_{q-e}+F_{q-i},$$

where *F_p_*_-*e*_ and *F_q_*_-*e*_ are the electrostatic forces, while *F_p_*_-*i*_ and *F_q_*_-_*_i_* are the inertia forces.

The inertia forces of a hemispherical resonator are described by

(24) $$F_{p-i}=-m_{h}a_{p},F_{q-i}=-m_{h}a_{q},$$

where *a_p_*, *a_q_*, and *m_h_* are the acceleration and equivalent mass of the resonator, respectively.

The equations of motion of a hemispherical resonator under the second-order vibration mode are written as

(25) $$\{\begin{array}{l} \ddot{p}+2c_{p}\dot{p}+\omega_{0}^{2}p=f_{p}+g_{p}\\ \ddot{q}+2c_{q}\dot{q}+\omega_{0}^{2}q=f_{q}+g_{q} \end{array},$$

where

(26) $$2c_{p}=\frac{\xi_{p}}{I_{0}},2c_{q}=\frac{\xi_{q}}{I_{0}},f_{p}=\frac{F_{p-e}}{I_{0}},f_{q}=\frac{F_{q-e}}{I_{0}},g_{p}=\frac{F_{p-i}}{I_{0}},g_{q}=\frac{F_{q-i}}{I_{0}},$$

where *f_p_* and *f_q_* are the electrostatic-specific forces, while *g_p_* and *g_q_* are the inertia-specific forces. Under ideal conditions, *c_p_* = *c_q_* = *c*.

(27) $$\omega_{0}=\sqrt{\frac{I_{1}}{I_{0}}}.$$

Equation (27) is the natural angular frequency of an ideal hemispherical resonator. Based on the basic parameters of the resonator in Table 1, the natural angular frequency *ω*_0_ = 12,393.6 rad/s, and its corresponding natural frequency *f*_0_ = 1972.5 Hz.

The relative damping coefficient of a hemispherical resonator is defined as follows [31]:

(28) $$\zeta =\frac{c}{\omega_{0}}.$$

The quality factor of a hemispherical resonator can be expressed as follows [31]:

(29) $$Q_{f}=\frac{1}{2\zeta }.$$

Substituting Equation (29) into Equation (28) yields

(30) $$2c=\frac{\omega_{0}}{Q_{f}}.$$

Applying Equation (30) into Equation (25), the equations of motion of a hemispherical resonator under the second-order vibration mode are finally written as

(31) $$\{\begin{array}{l} \ddot{p}+\frac{\omega_{0}}{Q_{f}}\dot{p}+\omega_{0}^{2}p=f_{p}+g_{p}\\ \ddot{q}+\frac{\omega_{0}}{Q_{f}}\dot{q}+\omega_{0}^{2}q=f_{q}+g_{q} \end{array}.$$

Equation (31) is the basis of the error mechanism analysis of a hemispherical resonator containing the first–third harmonics of mass imperfection under linear vibration excitation.

## 3. Effect of Linear Vibration on Standing Wave of Imperfect Hemispherical Resonator

In this section, the binding problems of a standing wave of a hemispherical resonator containing the first–third harmonics of mass imperfection are analyzed under linear vibration excitation.

### 3.1. Effect of Vertical Linear Vibration on Standing Wave of Imperfect Hemispherical Resonator

As shown in Figure 2, the hemispherical resonator is subjected to linear vibrations in the *x-*, *y-*, and *z*-directions. The first choice is to analyze the influence of vertical linear vibration on the standing wave of a hemispherical resonator.

The linear vibration of a hemispherical resonator in the *z_h_* axis direction is expressed as

(32) $$z=z_{0}\cos\lambda t,$$

where *z*_0_ and *λ* represent the vibration amplitude and frequency of the resonator, respectively.

The linear acceleration in the *z_h_* axis direction is obtained by

(33) $$a_{z}=-z_{0}\lambda^{2}\cos\lambda t.$$

The linear acceleration in the *z_h_* axis direction is projected into the local coordinate system *P*-*e*_1_*e*_2_*m* as

(34) $$Z_{e}=C_{h}^{e}{\left[\begin{array}{ccc} 0 & 0 & a_{z} \end{array}\right]}^{T}={\left[\begin{array}{ccc} -a_{z}\sin\alpha & 0 & a_{z}\cos\alpha \end{array}\right]}^{T}.$$

The equations of motion of a hemispherical resonator under linear vibration excitation in the *z_h_* axis direction are given by

(35) $$\{\begin{array}{l} \ddot{p}+\frac{\omega_{0}}{Q_{f}}\dot{p}+\omega_{0}^{2}p=-\frac{1}{I_{0}}\int_{R-\frac{h}{2}}^{R+\frac{h}{2}}\int_{0}^{2\pi }\int_{0}^{\frac{\pi }{2}}Z_{e}^{T}H_{p}\rho \left(\beta \right)r^{2}\sin\alpha drd\alpha d\beta \\ \ddot{q}+\frac{\omega_{0}}{Q_{f}}\dot{q}+\omega_{0}^{2}q=-\frac{1}{I_{0}}\int_{R-\frac{h}{2}}^{R+\frac{h}{2}}\int_{0}^{2\pi }\int_{0}^{\frac{\pi }{2}}Z_{e}^{T}H_{q}\rho \left(\beta \right)r^{2}\sin\alpha drd\alpha d\beta \end{array},$$

where

(36) $$\{\begin{array}{l} H_{p}={\left[\begin{array}{ccc} U\cos2\beta & V\sin2\beta & W\cos2\beta \end{array}\right]}^{T}\\ H_{q}={\left[\begin{array}{ccc} U\sin2\beta & -V\cos2\beta & W\sin2\beta \end{array}\right]}^{T} \end{array}.$$

Substituting Equations (14), (34), and (36) into Equation (35) yields

(37) $$\{\begin{array}{l} \ddot{p}+\frac{\omega_{0}}{Q_{f}}\dot{p}+\omega_{0}^{2}p=\frac{G}{M}\varepsilon_{2}z_{0}\lambda^{2}\cos2\beta_{2}\cos\lambda t\\ \ddot{q}+\frac{\omega_{0}}{Q_{f}}\dot{q}+\omega_{0}^{2}q=\frac{G}{M}\varepsilon_{2}z_{0}\lambda^{2}\sin2\beta_{2}\cos\lambda t \end{array},$$

where

(38) $$G=\int_{0}^{\frac{\pi }{2}}\left[W\left(\alpha \right)\cos\alpha -U\left(\alpha \right)\sin\alpha \right]\sin\alpha d\alpha ,$$

(39) $$M=\int_{0}^{\frac{\pi }{2}}\left[U^{2}\left(\alpha \right)+V^{2}\left(\alpha \right)+W^{2}\left(\alpha \right)\right]\sin\alpha d\alpha .$$

The steady-state response of Equation (36) can be represented by

(40) $$\{\begin{array}{l} p\left(t\right)=a\cos\lambda t+m\sin\lambda t\\ q\left(t\right)=b\cos\lambda t+n\sin\lambda t \end{array},$$

where *a*, *m*, *b*, and *n* are the amplitude parameters of the steady-state response of the vibration system.

Substituting Equation (40) into Equation (37), when *λ* = *ω*_0_, the parameters of vibration amplitude are obtained by

(41) $$\{\begin{array}{l} \left[\begin{array}{c} m\\ n \end{array}\right]=Q_{f}\frac{G}{M}z_{0}\left[\begin{array}{l} \varepsilon_{2}\cos2\beta_{2}\\ \varepsilon_{2}\sin2\beta_{2} \end{array}\right]\\ a=b=0 \end{array},$$

where *G*/*M* = −0.4 on the basis of the parameters in Table 1.

**Figure 5:**
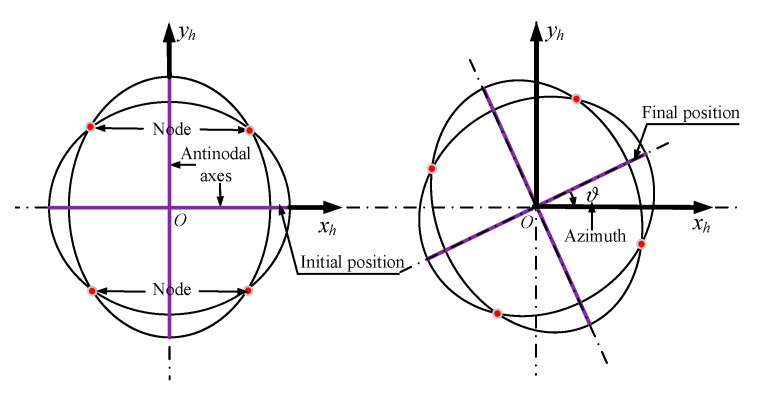
Azimuth of a standing wave.

Substituting Equation (40) into Equation (8), the radial vibration of a hemispherical resonator is expressed as

(42) $$w\left(\alpha ,\beta ,t\right)=W\left(\alpha \right)\sqrt{m^{2}+n^{2}}\cos2\left(\beta -\vartheta \right)\sin\omega_{0}t,$$

where

(43) $$\tan2\vartheta =\frac{n}{m},$$

where *ϑ* represents the azimuth of the antinode of the standing wave relative to the initial position, as shown in Figure 5.

Substituting Equation (41) into Equation (43), the azimuth of a standing wave is obtained by

(44) $$\tan2\vartheta =\tan2\beta_{2}.$$

Equation (44) shows that the standing wave is bound in the orientation of the second harmonic of mass imperfection under linear vibration in the *z_h_* axis direction.

Substituting Equations (41) and (40) into Equation (8), the steady-state radial vibration is obtained by

(45) $$w_{z}\left(\alpha ,\beta ,t\right)=W\left(\alpha \right)Q_{f}\frac{G}{M}z_{0}\sin\omega_{0}t\left[\varepsilon_{2}\cos2\left(\beta -\beta_{2}\right)\right].$$

### 3.2. Effect of Horizonal Linear Vibration on Standing Wave of Imperfect Hemispherical Resonator

After analyzing the influence of vertical vibration, the next step is to demonstrate the influence of horizontal vibration on the standing wave of a resonator, where the horizontal linear vibration consists of two directions: *x_h_* and *y_h_*.

The linear vibration of a hemispherical resonator in the *x_h_* axis direction is expressed as

(46) $$x=x_{0}\cos\lambda t,$$

where *x*_0_ and *λ* represent the vibration amplitude and frequency of the resonator, respectively.

The linear acceleration of a hemispherical resonator in the *x_h_* axis direction is obtained by

(47) $$a_{x}=-x_{0}\lambda^{2}\cos\lambda t.$$

The linear acceleration in the *x_h_* axis direction is projected into the local coordinate system *P*-*e*_1_*e*_2_*m* as

(48) $$X_{e}=C_{h}^{e}{\left[\begin{array}{ccc} a_{x} & 0 & 0 \end{array}\right]}^{T}={\left[\begin{array}{ccc} a_{x}\cos\beta \cos\alpha & -a_{x}\sin\beta & a_{x}\cos\beta \sin\alpha \end{array}\right]}^{T}.$$

The equations of motion of a hemispherical resonator under linear vibration excitation in the *x_h_* axis direction are given by

(49) $$\{\begin{array}{l} \ddot{p}+\frac{\omega_{0}}{Q_{f}}\dot{p}+\omega_{0}^{2}p=-\frac{1}{I_{0}}\int_{R-\frac{h}{2}}^{R+\frac{h}{2}}\int_{0}^{2\pi }\int_{0}^{\frac{\pi }{2}}X_{e}^{T}H_{p}\rho \left(\beta \right)r^{2}\sin\alpha drd\alpha d\beta \\ \ddot{q}+\frac{\omega_{0}}{Q_{f}}\dot{q}+\omega_{0}^{2}q=-\frac{1}{I_{0}}\int_{R-\frac{h}{2}}^{R+\frac{h}{2}}\int_{0}^{2\pi }\int_{0}^{\frac{\pi }{2}}X_{e}^{T}H_{q}\rho \left(\beta \right)r^{2}\sin\alpha drd\alpha d\beta \end{array}.$$

Substituting Equations (14), (36), and (48) into Equation (49) yields

(50) $$\{\begin{array}{l} \ddot{p}+\frac{\omega_{0}}{Q_{f}}\dot{p}+\omega_{0}^{2}p=\frac{1}{M}\lambda^{2}x_{0}\cos\lambda t[\left(A-B+C\right)\varepsilon_{1}\cos\beta_{1}+\left(A+B+C\right)\varepsilon_{3}\cos3\beta_{3}]\\ \ddot{q}+\frac{\omega_{0}}{Q_{f}}\dot{q}+\omega_{0}^{2}q=\frac{1}{M}\lambda^{2}x_{0}\cos\lambda t[\left(A-B+C\right)\varepsilon_{1}\sin\beta_{1}+\left(A+B+C\right)\varepsilon_{3}\sin3\beta_{3}] \end{array},$$

where

(51) $$A=\int_{0}^{\frac{\pi }{2}}U\left(\alpha \right)\sin\alpha \cos\alpha d\alpha ,$$

(52) $$B=\int_{0}^{\frac{\pi }{2}}V\left(\alpha \right)\sin\alpha d\alpha ,$$

(53) $$C=\int_{0}^{\frac{\pi }{2}}W\left(\alpha \right)\sin^{2}\alpha d\alpha .$$

According to Equations (51)–(53), it can be calculated that

(54) $$\frac{A+B+C}{A-B+C}=\frac{1}{3}.$$

Substituting Equation (54) into Equation (50) yields

(55) $$\{\begin{array}{l} \ddot{p}+\frac{\omega_{0}}{Q_{f}}\dot{p}+\omega_{0}^{2}p=\frac{A+B+C}{M}\lambda^{2}x_{0}\cos\lambda t\left(3\varepsilon_{1}\cos\beta_{1}+\varepsilon_{3}\cos3\beta_{3}\right)\\ \ddot{q}+\frac{\omega_{0}}{Q_{f}}\dot{q}+\omega_{0}^{2}q=\frac{A+B+C}{M}\lambda^{2}x_{0}\cos\lambda t\left(3\varepsilon_{1}\sin\beta_{1}+\varepsilon_{3}\sin3\beta_{3}\right) \end{array}.$$

Substituting Equation (40) into Equation (55), when *λ* = ω_0_, the parameters of vibration amplitude are obtained by

(56) $$\{\begin{array}{l} \left[\begin{array}{c} m\\ n \end{array}\right]=Q_{f}\frac{A+B+C}{M}x_{0}\left[\begin{array}{l} 3\varepsilon_{1}\cos\beta_{1}+\varepsilon_{3}\cos3\beta_{3}\\ 3\varepsilon_{1}\sin\beta_{1}+\varepsilon_{3}\sin3\beta_{3} \end{array}\right]\\ a=b=0 \end{array},$$

where (*A + B + C*)/*M* = −0.23 on the basis of applying the parameters in Table 1.

Substituting Equation (56) into Equation (43), the azimuth of a standing wave is obtained by

(57) $$\tan2\vartheta =\frac{3\varepsilon_{1}\sin\beta_{1}+\varepsilon_{3}\sin3\beta_{3}}{3\varepsilon_{1}\cos\beta_{1}+\varepsilon_{3}\cos3\beta_{3}}.$$

Equation (57) shows that the standing wave is bound in the orientation of the first and third harmonics of mass imperfection under linear vibration excitation in the *x_h_* axis direction.

Substituting Equations (56) and (40) into Equation (8), the steady-state radial vibration of a resonator is given by

(58) $$w_{x}\left(\alpha ,\beta ,t\right)=W\left(\alpha \right)Q_{f}\frac{A+B+C}{M}x_{0}\sin\omega_{0}t\left[3\varepsilon_{1}\cos2\left(\beta -\frac{\beta_{1}}{2}\right)+\varepsilon_{3}\cos2\left(\beta -\frac{3\beta_{3}}{2}\right)\right].$$

The linear vibration of a hemispherical resonator in the *y_h_* axis direction is expressed as

(59) $$y=y_{0}\cos\lambda t,$$

where *y*_0_ and *λ* represent the vibration amplitude and frequency of the resonator, respectively.

The linear acceleration in the *y_h_* axis direction is obtained by

(60) $$a_{y}=-y_{0}\lambda^{2}\cos\lambda t.$$

The linear acceleration in the *y_h_* axis direction is projected into the local coordinate system *P*-*e*_1_*e*_2_*m* as

(61) $$Y_{e}=C_{h}^{e}{\left[\begin{array}{ccc} 0 & a_{y} & 0 \end{array}\right]}^{T}={\left[\begin{array}{ccc} a_{y}\sin\beta \cos\alpha & a_{y}\cos\beta & a_{y}\sin\beta \sin\alpha \end{array}\right]}^{T}.$$

The equations of motion of a hemispherical resonator under linear vibration excitation in the *y_h_* axis direction are given by

(62) $$\{\begin{array}{l} \ddot{p}+\frac{\omega_{0}}{Q_{f}}\dot{p}+\omega_{0}^{2}p=-\frac{1}{I_{0}}\int_{R-\frac{h}{2}}^{R+\frac{h}{2}}\int_{0}^{2\pi }\int_{0}^{\frac{\pi }{2}}Y_{e}^{T}H_{p}\rho \left(\beta \right)r^{2}\sin\alpha drd\alpha d\beta \\ \ddot{q}+\frac{\omega_{0}}{Q_{f}}\dot{q}+\omega_{0}^{2}q=-\frac{1}{I_{0}}\int_{R-\frac{h}{2}}^{R+\frac{h}{2}}\int_{0}^{2\pi }\int_{0}^{\frac{\pi }{2}}Y_{e}^{T}H_{q}\rho \left(\beta \right)r^{2}\sin\alpha drd\alpha d\beta \end{array}.$$

Substituting Equations (14), (36), and (61) into Equation (62) yields

(63) $$\{\begin{array}{l} \ddot{p}+\frac{\omega_{0}}{Q_{f}}\dot{p}+\omega_{0}^{2}p=\frac{A+B+C}{M}y_{0}\lambda^{2}\cos\lambda t\left(-3\varepsilon_{1}\sin\beta_{1}+\varepsilon_{3}\sin3\beta_{3}\right)\\ \ddot{q}+\frac{\omega_{0}}{Q_{f}}\dot{q}+\omega_{0}^{2}q=\frac{A+B+C}{M}y_{0}\lambda^{2}\cos\lambda t\left(3\varepsilon_{1}\cos\beta -\varepsilon_{3}\cos3\beta_{3}\right) \end{array}.$$

Substituting Equation (40) into Equation (63), when *λ* = *ω*_0_, the parameters of vibration amplitude are obtained by

(64) $$\{\begin{array}{l} \left[\begin{array}{c} m\\ n \end{array}\right]=Q_{f}\frac{A+B+C}{M}y_{0}\left[\begin{array}{l} -3\varepsilon_{1}\sin\beta_{1}+\varepsilon_{3}\sin3\beta_{3}\\ 3\varepsilon_{1}\cos\beta_{1}-\varepsilon_{3}\cos3\beta_{3} \end{array}\right]\\ a=b=0 \end{array}.$$

Substituting Equation (64) into Equation (43), the azimuth of a standing wave is obtained by

(65) $$\tan2\vartheta =\frac{3\varepsilon_{1}\cos\beta_{1}-\varepsilon_{3}\cos3\beta_{3}}{-3\varepsilon_{1}\sin\beta_{1}+\varepsilon_{3}\sin3\beta_{3}}.$$

Equation (65) shows that the standing wave is bound in the orientation of the first and third harmonics of mass imperfection under linear vibration excitation in the *y_h_* axis direction.

Substituting Equations (64) and (40) into Equation (8), the steady-state radial vibration of a resonator is expressed by

(66) $$w_{y}\left(\alpha ,\beta ,t\right)=W\left(\alpha \right)Q_{f}\frac{A+B+C}{M}y_{0}\sin\omega_{0}t\left[3\varepsilon_{1}\sin2\left(\beta -\frac{\beta_{1}}{2}\right)+\varepsilon_{3}\sin2\left(\beta -\frac{3\beta_{3}}{2}\right)\right].$$

## 4. Identification of Location of First–Third Harmonics of Mass Imperfection by Linear Vibration

In this section, the identification method for the locations of the first–third harmonics of mass imperfection of a hemispherical resonator by vertical and horizontal vibration is proposed.

### 4.1. Identification for Location of the Second Harmonic of Mass Imperfection by Vertical Vibration

Under vertical linear vibration excitation, the radial vibration of the bottom (*α* = π/2) of a resonator at *β* = 0 and *β* = π/4 can be expressed by

(67) $$\{\begin{array}{c} w_{z}^{*}\left(\frac{\pi }{2},0,t\right)=W\left(\frac{\pi }{2}\right)Q_{f}\frac{G}{M}z_{0}\sin\omega_{0}t\varepsilon_{2}\cos2\beta_{2}\\ w_{z}^{*}\left(\frac{\pi }{2},\frac{\pi }{4},t\right)=W\left(\frac{\pi }{2}\right)Q_{f}\frac{G}{M}z_{0}\sin\omega_{0}t\varepsilon_{2}\sin2\beta_{2} \end{array}.$$

The azimuth of the second harmonic of mass imperfection of a resonator is obtained by

(68) $$\tan2\beta_{2}=\frac{w_{z}^{*}\left(\frac{\pi }{2},\frac{\pi }{4},t\right)}{w_{z}^{*}\left(\frac{\pi }{2},0,t\right)}.$$

### 4.2. Calibration for Locations of the First and Third Harmonics of Mass Imperfection by Horizontal Vibration

Under horizontal linear vibration excitation, the radial vibration of the bottom (*α* = π/2) of a resonator at *β* = 0 and *β* = π/4 can be expressed as

(69) $$\{\begin{array}{c} w_{x}^{*}\left(\frac{\pi }{2},0,t\right)=W\left(\frac{\pi }{2}\right)Q_{f}\frac{A+B+C}{M}x_{0}\sin\omega_{0}t\left(3\varepsilon_{1}\cos\beta_{1}+\varepsilon_{3}\cos3\beta_{3}\right)\\ w_{x}^{*}\left(\frac{\pi }{2},\frac{\pi }{4},t\right)=W\left(\frac{\pi }{2}\right)Q_{f}\frac{A+B+C}{M}x_{0}\sin\omega_{0}t\left(3\varepsilon_{1}\sin\beta_{1}+\varepsilon_{3}\sin3\beta_{3}\right) \end{array}.$$

In order to identify the locations of the first and third harmonics of mass imperfection, the resonator is rotated 90°; then, the radial vibration of the resonator under horizontal linear vibration excitation can be described by

(70) $$w_{x}\left(\alpha ,\beta ,t\right)=W\left(\alpha \right)Q_{f}\frac{A+B+C}{M}x_{0}\sin\omega_{0}t\left[3\varepsilon_{1}\cos\left(2\beta -\beta_{1}-\frac{\pi }{2}\right)+\varepsilon_{3}\cos\left(2\beta -3\beta_{3}-\frac{3\pi }{2}\right)\right].$$

Similarly, according to Equation (70), the radial vibration of the bottom (α = π/2) of a resonator at *β* = 0 and *β* = π/4 can be expressed as

(71) $$\{\begin{array}{c} w_{x_{2}}^{*}\left(\frac{\pi }{2},0,t\right)=W\left(\frac{\pi }{2}\right)Q_{f}\frac{A+B+C}{M}x_{0}\sin\omega_{0}t\left(-3\varepsilon_{1}\sin\beta_{1}+\varepsilon_{3}\sin3\beta_{3}\right)\\ w_{x_{2}}^{*}\left(\frac{\pi }{2},\frac{\pi }{4},t\right)=W\left(\frac{\pi }{2}\right)Q_{f}\frac{A+B+C}{M}x_{0}\sin\omega_{0}t\left(3\varepsilon_{1}\cos\beta_{1}-\varepsilon_{3}\cos3\beta_{3}\right) \end{array}.$$

By solving Equations (69) and (71), the azimuth of the first harmonic of mass imperfection of a resonator is given by

(72) $$\tan\beta_{1}=\frac{w_{x}^{*}\left(\frac{\pi }{2},\frac{\pi }{4},t\right)-w_{x_{2}}^{*}\left(\frac{\pi }{2},0,t\right)}{w_{x}^{*}\left(\frac{\pi }{2},0,t\right)+w_{x_{2}}^{*}\left(\frac{\pi }{2},\frac{\pi }{4},t\right)}.$$

Similarly, the azimuth of the third harmonic of mass imperfection of a resonator is obtained by

(73) $$\tan3\beta_{3}=\frac{w_{x}^{*}\left(\frac{\pi }{2},\frac{\pi }{4},t\right)+w_{x_{2}}^{*}\left(\frac{\pi }{2},0,t\right)}{w_{x}^{*}\left(\frac{\pi }{2},0,t\right)-w_{x_{2}}^{*}\left(\frac{\pi }{2},\frac{\pi }{4},t\right)}.$$

## 5. Numerical Simulation Results

Here, the standing wave binding phenomenon of a hemispherical resonator containing the first–third harmonics of mass imperfection and the location identification method of mass imperfection are verified using a numerical simulation model of the vibration system, which is based on the equations of motion and radial vibration displacement of a resonator.

The radial displacement detection of a resonator is usually completed by two detection electrodes, which are positioned at 0° and 45° in the circumferential direction of the resonator, as shown in Figure 4. The surfaces of both electrodes are metalized with a thin platinum coating. Similarly, the inner surface of the resonator is also metallized with a thin platinum coating. A capacitor is formed between the resonator and electrodes. Thus, the change in radial vibration displacement of the resonator can be reflected by the change in capacitance.

The signals detected by sensors 1 and 2 can be expressed by

(74) $$w_{1}^{}=W\left(\frac{\pi }{2}\right)m\sin\lambda t,w_{2}^{}=W\left(\frac{\pi }{2}\right)n\sin\lambda t.$$

The output signals of the displacement detection electrodes can be demodulated by the reference signal sin*λt*; then, the following equation can be obtained:

(75) $$\tan2\vartheta =\frac{w_{2}^{*}}{w_{1}^{*}},$$

where $w_{1}^{*}$ and $w_{2}^{*}$ represent the demodulated signals.

The demodulated signals need to be filtered by the low-pass filter, for which the order and pass band edge frequency are set to 2 and 20 rad/s in the numerical simulation model of the vibration system, respectively.

**Figure 6:**
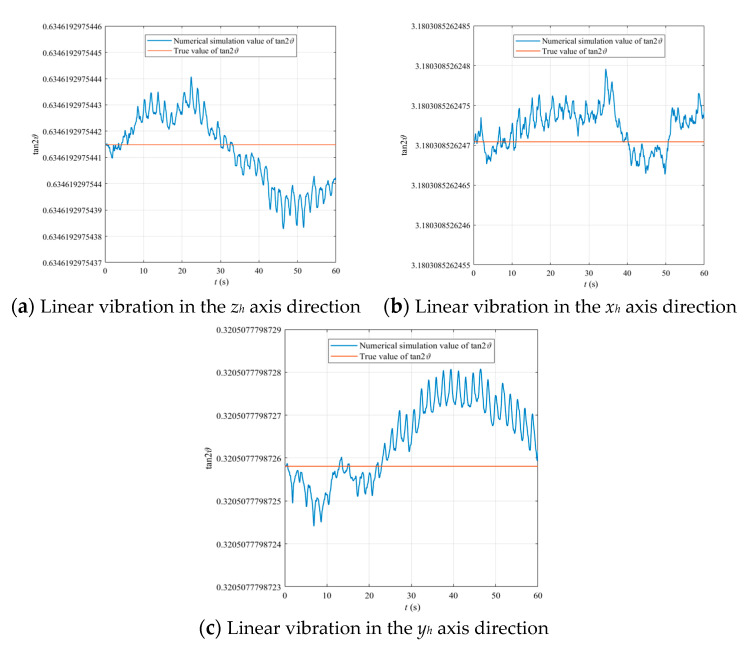
Standing wave binding of a hemispherical resonator containing the first–third harmonics of mass imperfection under linear vibration.

**Figure 7:**
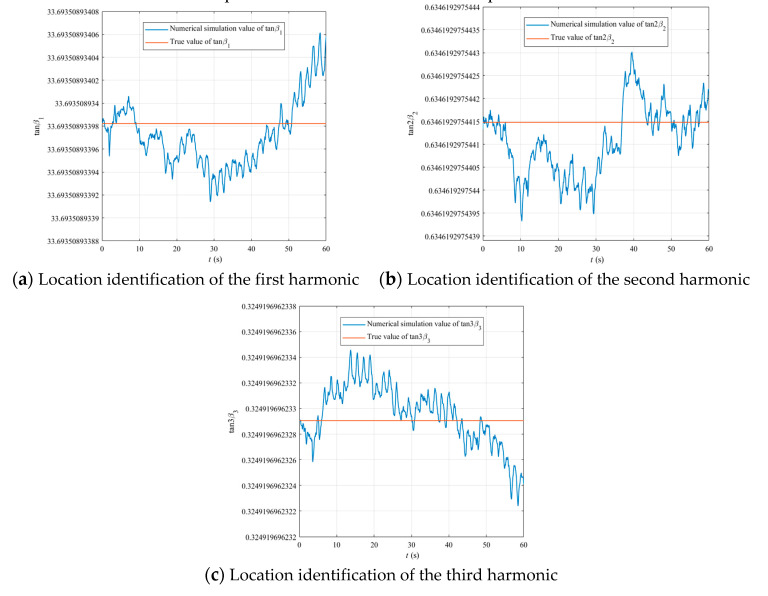
Location identification of the first–third harmonics of mass imperfection of a hemispherical resonator under linear vibration.

The basic parameters of the numerical simulation model are as follows: *x*_0_ = *y*_0_ = *z*_0_ = 1 mm, *ε*_1_ = *ε*_2_ = *ε*_3_ = 10^−6^, *β*_1_ = 88.3°, *β*_2_ = 16.2°, *β*_3_ = 6.0°, *λ* = *ω*_0_ = 1972.5 Hz, *Q_f_* = 10^6^, *G*/*M* = −0.4, (*A + B + C*)/*M* = −0.23. The numerical simulation results are shown in Figure 6 and Figure 7.

Figure 6a–c indicate the correctness of the azimuth of the standing wave derived from the equations of motion of a hemispherical resonator with mass imperfection under linear vibration.

As shown in Figure 7a–c, the numerical simulation model based on the equations of motion of an imperfect resonator can verify the correctness of the identification method for the locations of the first–third harmonics of mass imperfection.

## 6. Conclusions

In order to analyze the influence of the first–third harmonics of mass imperfection on the vibration characteristics of a hemispherical resonator, the equations of motion of a resonator containing the first–third harmonics of mass imperfection under linear vibration excitation were established using the elastic thin shell theory and the Lagrange mechanics principle. Through dynamic analysis, it can be concluded that, when the frequency of external linear vibration is equal to the natural frequency of the resonator, the standing wave under vertical linear vibration excitation is bound in the azimuth of the second harmonic of mass imperfection; similarly, the standing wave is bound in the azimuth of the first and third harmonics of mass imperfection under horizontal linear vibration excitation, which indicates that linear vibration excitation can cause a parasitic component to be hidden in the azimuth of the standing wave, which can cause the output signal distortion of the gyroscope. Therefore, it is very important to identify the position of the first - third harmonics of mass imperfection. According to the binding phenomenon of standing wave, vertical linear vibration can be used to calibrate the location of the second harmonic of mass imperfection, and horizontal linear vibration can be used to determine the locations of the first and third harmonics of mass imperfection, which can provide a theoretical method for the mass balance of an imperfect resonator.

## Figures and Tables

**Table 1:** Geometric and physical parameters of resonator made of fused quartz.

Symbol	Name	Value	Symbol	Name	Value
*ρ*_0_	Average density	2500 kg/m^3^	*l*	Length of supporting column	60 mm
*E*	Young’s modulus	76.7 GPa	*r*_1_	Radius of supporting column	6 mm
*μ*	Poisson’s ratio	0.17	*R*	Radius of mid-surface	25 mm
*Q_f_*	Quality factor	10^6^	*R*_3_	Inner corner radius	3 mm
*h*	Thickness	1 mm	*R*_4_	Outer corner radius	3 mm
